# Effect of Active Video Games on Healthy Children’s Fundamental Motor Skills and Physical Fitness: A Systematic Review

**DOI:** 10.3390/ijerph17218264

**Published:** 2020-11-09

**Authors:** Wenxi Liu, Nan Zeng, Daniel J. McDonough, Zan Gao

**Affiliations:** 1 School of Kinesiology, University of Minnesota-Twin Cities, Minneapolis, MN 55455, USA; liux4443@umn.edu (W.L.); mcdo0785@umn.edu (D.J.M.); 2 Prevention Research Center, Department of Pediatrics, School of Medicine, University of New Mexico, Albuquerque, NM 87131, USA; NZeng@salud.unm.edu

**Keywords:** children, exergaming, locomotor skills, object control skills

## Abstract

*Objective*: The present study aimed to synthesize the most updated literature regarding the casual evidence of the effects of active video games (AVGs) on fundamental motor skills (FMS; locomotor skills and object control skills) and physical fitness among healthy children. *Methods*: Electronic databases were searched through October 2020. Peer-reviewed randomized control trials (RCTs) and quasi-experimental designs examining the effectiveness of AVGs on FMS and physical fitness development among healthy children (3–12 years) were screened. *Results*: A total of nine RCTs and one quasi-experimental study were included. Of the five studies examining the effect of AVGs on FMS, two reported significant improvements, while three reported no significant improvements in motor skills development as compared to control. Of the five studies assessing the effects of AVGs on physical fitness, four reported significant improvements in physical fitness such as balance, agility, and speed, whereas one reported significant improvements in skill-related executive function, but not in physical competence. *Conclusions*: Overall, the current available evidence supports AVGs as an effective means to improve physical fitness, such as balance, postural stability, and agility, among healthy children. However, the findings of AVGs on healthy children’s object control and locomotor skills remain inconclusive.

## 1. Introduction

The development of fundamental motor skills (FMS) has been considered as the foundation of competently performing different types of physical activity (PA), as children need to first develop these essential movement skills to most effectively engage in PA behaviors [1]. In general, children’s FMS are developed through various types of PA [2,3]. Indeed, previous research has indicated FMS development to be a key determinant in promoting PA participation, physical fitness, and other health-related outcomes as these motor skills are the basis for performing more complex PA in the future [4]. In detail, FMS requires the activation of large muscle groups and are generally dichotomized into locomotor skills and object control skills [5]. Locomotor skills include running, jumping, hopping, leaping, galloping, and sliding as different movements to transport the body from one location to another, whereas object control skills involve the transporting, intercepting, or projecting of objects, such as throwing, catching, dribbling, kicking, underhand rolling, and striking [6]. In addition, physical fitness, such as balance, coordination, agility, reaction time, and speed, also facilitates the development of overall FMS because physical fitness indicates the body’s capability and competence of performing quality motor skills [7]. Notably, previous studies have also included balance and stability—measures of physical fitness—as components of FMS [1,8]. Typically, FMS are initialized and developed through various movement activities during early childhood as children develop competency for those simple FMS tasks, and later, more complex skills will be developed and mastered by participating in different types of PA [9]. However, children with inadequate motor skills may experience developmental delays as they get older, which may decrease their PA participation [10]. Thus, it is crucial to adequately develop children’s FMS and physical fitness during childhood, which allows them to independently navigate their environment and contribute to their overall health and wellbeing [11,12,13]. Given that children spend most of their time in school or childcare centers, physical education programs offer opportunities for them to be physically active and to develop necessary motor skills by engaging in various PAs. However, children often do not develop their motor skills proficiently through physical education, likely due to limited class time and overcrowded classes, which is not conducive to developing FMS in this population [14]. Compiling research has indicated that there is an increasing number of children who no longer possess proficient FMS and who are not capable of hopping, kicking, skipping, throwing, or running with proper form [15,16]. Indeed, Hardy et al. [16] conducted a longitudinal study to investigate children’s and adolescents’ FMS development trends across 13 years, with findings indicating that less than 50% children and adolescents demonstrated FMS competency. Similarly, recent cross-sectional evidence observed that approximately 77% of children are at-risk for developmental delay [17]. In addition to conventional physical education programs, an innovative and fun approach to improve FMS and physical fitness may be through active video games (AVGs) given their requirement for gross motor activity to play.

Active video games (AVGs) have been suggested to be an effective strategy for promoting PA among children and adolescents [18]. In detail, AVGs combine video games and exercise, thereby motivating players to engage in PA while playing the games [19]. In order to play AVGs, players need to make a physical effort to interact with the gaming environment by using their upper and lower extremities to perform various activities, such as jumping, catching, dancing, and jogging [20]. In recent years, an increasing number of studies have attempted to adopt this fun and innovative approach for promoting PA and health among various populations. Promising findings suggest that AVGs may be an effective alternative in promoting PA and providing comparable health benefits as conventional PA strategies in children and adolescents [21,22]. Given the important role of FMS and physical fitness in child development, recent studies have started to investigate the effects of AVGs on motor skills competence and preliminary findings have indicated the potential to improve FMS and physical fitness in children with various clinical conditions [14,23,24,25]. For example, Gao et al. [23] conducted an 8-week AVG-based intervention among preschool children and found statistically significantly greater increases in PA among intervention children, supporting its potential for enhancing children’s motor skills competence. Moreover, Vernadakis et al. [26] conducted an 8-week AVG-based intervention and observed greater improvement in object control skills in the AVG group compared to control, further supporting the evidence that the use of AVGs could be a feasible and fun approach for improving FMS among elementary school children. However, there were also studies that found no impact of AVGs on children’s FMS development [27,28]. For example, Barnett et al. [27] conducted a 6-week randomized controlled trial (RCT) and found that object control skills improved over time but there were no group differences regarding FMS. Notably, the investigators postulated that these findings were attributed to a lack of playing instructions during the intervention and a low intervention dose of only six weeks.

Although increasing findings support AVGs as a favorable approach to improving children’s FMS and physical fitness, the effectiveness of AVG-based interventions remains unclear and the findings are mixed. Recently, an increasing number of high-quality studies (e.g., RCTs) have employed AVGs as an intervention strategy by which to facilitate the development of FMS and physical fitness among children; thus, an updated comprehensive review regarding their utility to improve children’s motor skills development is needed. Therefore, the purpose of this study was to systematically synthesize the most recent literature and provide an updated review to summarize the empirical findings regarding the impact of AVGs on healthy children’s FMS and physical fitness.

## 2. Methods

This systematic review followed the Preferred Reporting Items for Systematic Reviews and Meta-Analyses (PRISMA-P) reporting guidelines [29].

### 2.1. Operational Definitions

Active video games (AVGs): a type of movement-based video game that is also a form of exercise given their requirement for gross motor activity [21,30].

Fundamental motor skills (FMS): considered as the “building blocks” of more advanced, complicated movements that are required to participate in sports, games, or other context-specific PAs, such as object control skills (e.g., throwing, catching dribbling, kicking, striking, underhand rolling) and locomotor skills (e.g., walking, running, jumping, hopping, leaping, galloping, sliding, skipping) [8].

Physical fitness: includes the components of agility, speed, coordination, power, reaction time, and balance which facilitate the body’s capability to perform activities of daily living [31].

### 2.2. Search Strategies

To ensure the inclusion of relevant literature, we conducted a comprehensive search to identify qualified studies. The following databases were searched to retrieve the literature: (1) Academic Search Complete; (2) Communication and Mass Media Complete; (3) ERIC; (4) PsycINFO; (5) PubMed; (6) SportDiscus; and (7) Medline. We also searched Google scholar. The following keywords were used in various logical combinations: (“active video game” OR “exergame” OR “exergaming” OR “Wii” OR “Kinect” OR “PlayStation”) AND (“fundamental movement skills” OR “motor development” OR “motor skills” OR “physical fitness” OR “physical competence” OR “skill-related fitness” OR “motor proficiency” OR “physical competence”) AND (“healthy children” OR “Children” OR “preschool children” OR “school children”). Only peer-reviewed articles published in English were included in the review. In the first stage of the literature search, titles and abstracts of identified articles were checked for relevance by 2 authors (WL, ZG). In the second stage, full-text articles were retrieved and considered for inclusion based on the inclusion criteria. In the final stage, the reference lists of retrieved full-text articles were checked for possible inclusion.

### 2.3. Inclusion and Exclusion Criteria

To be included in this review, we applied the following criteria to screen for qualified studies: (1) published as peer-reviewed RCTs or quasi-experimental studies; (2) published in English between January 2010 and October 2020; (3) studied some type of AVG (e.g., Nintendo Wii, Xbox 360 Kinect, PlayStation, etc.); (4) targeted healthy children and children with overweight or obesity (aged 3–12 years); and (5) employed at least one motor skill or physical fitness assessment (e.g., running, siding, galloping, hopping, leaping, dodging, rolling, skipping, throwing, catching, kicking, jumping, stationary dribbling) and physical fitness assessment (e.g., static and dynamic balancing, reaction time, physical perception,).

The exclusion criteria included: (1) papers that were commentary articles, conference proceedings, and professional reports; (2) games that were not intended for home use or in an educational setting, such as arcade games and virtual reality-based sedentary computer games were not included; and (3) research that focused on special populations (e.g., physical disability, developmental coordination disorder, autism, etc.).

### 2.4. Data Extraction

Two reviewers (WL, NZ) separately screened the included articles and extracted the data following PRISMA standards [32]. If the reviewers were unable to determine the relevance of an article, then the abstract was reviewed. Data extraction was completed by one investigator (WL) and checked by two others (NZ, DJM) for accuracy. All potential articles were downloaded as full-text and stored in a shared folder, after which two authors (WL, NZ) reviewed each article independently to ensure that only relevant entries were included. A list of published articles on the topic was then created in a Microsoft Excel spreadsheet (Microsoft Corporation, Redmond, WA, USA). The following information was extracted: (1) year of publication and country of origin; (2) purpose of studies; (3) participant demographics (e.g., age); (4) methodological details (e.g., study design, study settings, intervention and control treatments, outcomes, AVG used, and measurement instruments); (5) key findings with respect to the potential of AVGs for individual’s motor skills promotion (e.g., reported changes in object control and/or locomotor skills, running speed, agility, balance and postural control, or other physical fitness measures).

Discrepancies between the investigators (WL, NZ) were discussed and resolved by consensus. Where a decision could not be reached, the corresponding author (ZG) reviewed the papers to make a final decision.

### 2.5. Quality and Risk of Bias in Individual Studies

In order to assess the risk of bias within the included studies, we adapted an assessment tool from previously published systematic reviews [19,33,34] which was developed based on Cochrane Collaboration’s tool [35]. The assessment included research designs, use of a power analysis for sample size, interpretation of data and results, etc. [36,37,38] (see Table 1). In detail, the study was marked as “+” when the study component was clearly described and presented, and when the study component was inadequate or missing, the study was recorded as “−”. Notably, two reviewers (WL, NZ) assessed the reviewed studies, respectively. When there were different scores between the two investigators, the third investigator (DJM) re-evaluated the studies. The final score for each study was calculated from the sum of “+” evaluations. The studies which were evaluated as “high quality and low risk of bias” were signified by a score greater than the median score of six, whereas “low quality and high risk for bias” studies were those which scored lower than the median score of six.

## 3. Results

**Figure 1:**
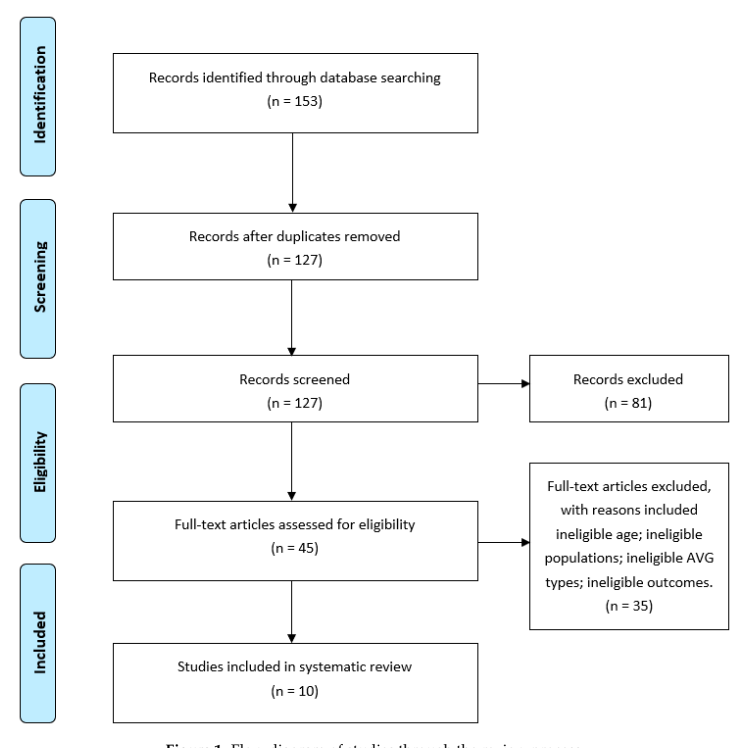
Flow diagram of studies through the review process.

A total of 153 potentially relevant articles were identified using the database searches. After removing duplicates, 127 peer-reviewed published studies were located. Following screening and cross-referencing the reference lists of included studies, 10 studies satisfied the preceding inclusion criteria (See Figure 1). Of the included studies, two studies (20%) targeted preschool children aged 3–5 years, seven studies (70%) targeted school children aged 6–12 years [25,26,27,39,40,41,43], and one study (10%) focused on children with overweight and obesity aged 9–12 years [31]. Among the included studies, eight RCTs (80%) and one quasi-experiment study (10%) were performed in school settings and one RCT (10%) was conducted in a laboratory setting [26]. The sample size ranged from 36 to 106 participants, and the intervention lengths ranged from 6 to 12 weeks, with an average of 6.8 weeks. With regard to AVGs used, eight studies (80%) employed commercially-available AVGs, such as Nintendo Wii and Xbox Kinect, and one study developed their own purpose-built AVG [25]. Overall, these articles were published during a period from January 2012 to May 2020 and across different countries. Specifically, two were from Australia [27,43], two from Canada [41,42], one from Turkey [39], one from U.S. [23], one from Greece [26], one from Ireland [25], one from China [43], and one from South Africa [31].

### 3.1. Quality and Risk of Bias Assessment

The risk assessment table for quality and bias among the included studies is shown in Table 1. There was more than 90% agreement between two reviewers (WL, NZ) in assessing the quality and risk of bias for each study. In detail, study quality ranged from four to seven points, with a median of six points. Eight (80%) of the included studies scored equal to or greater than the median score of six and were therefore considered high quality, while one (10%) of the included studies scored lower than the median score of six and was therefore considered low quality. The majority of the articles retained at least 70% of the participants and the measurement tools in all articles were valid. However, only five articles accounted for the analysis of missing values, four articles employed a power analysis prior to the experiment, and no articles reported six-month, post-intervention follow-up. The low scores were attributed to missing data, the absence of a power analysis, and a lack of follow-up. Notably, it was not feasible to conduct a meta-analysis due to the heterogeneity of the assessments across studies (e.g., RCTs and quasi-experiment designs make it difficult to conduct a meta-analysis). In addition, among the 10 included studies, five studies included at least one FMS component and the other five studies examined physical fitness, such as postural stability, agility, reaction time, and physical competence, whereas the measurement tools were different across studies. Lastly, the limited number of available RCTs may not have had enough power to conduct a meaningful meta-analysis.

### 3.2. Outcome Measures and Measurement Tools

Regarding relevant motor skills outcomes, six studies examined motor skills competence or motor skills-related self-perceptions [23,25,27,39,42,43], while three studies examined object control skills [26,27,40], two studies examined postural stability [40,41], one study examined locomotor skills [25], and one study examined agility (i.e., reaction time) [39].

Regarding the outcome assessments of the included studies, all studies employed appropriate and valid measurements and carried them out with standard protocols. For example, FMS were measured by the Test of Gross Motor Development (TGMD), which was most commonly used to evaluate competency of object control skills and locomotor skills. Regarding postural stability, a sophisticated portable assessment device (HUR BT4™) was used to test children’s balance. Briefly, postural stability was tested using six methods: single-leg and tandem stance each with eyes open or closed eyes on a hard surface and each stance with eyes open on a foam surface. One study assessed children’s agility in the form of measuring reaction times [39]. In detail, children were given directions to press the device button immediately after they received the auditory or visual stimuli and the reaction times were recorded using a timer. Regarding physical perception, one study used the Youth Physical Self Perception Profile (CY-PSPP), which was used to assess children’s perception of competence in sports competence, physical condition competence, and strength competence [39]. In addition, another study used the Pictorial Scale of Perceived Competence to assess preschool children’s perceived physical competence [42].

### 3.3. Fundamental Motor Skills (Object Control and Locomotor Skills)

A total of five studies examined one or both object control and locomotor skills, with three studies targeting object control skills [26,27,43], one study targeting locomotor skills [25], and one study targeting both locomotor and object control skills [23] (See Table A1). Overall, the findings regarding the effectiveness of AVGs on healthy children’s FMS remain inconclusive as three studies reported no significant between-group differences [23,27,43] and two studies found significant between-group differences [25,26]. Specifically, two studies examined the effects of AVGs (Nintendo Wii or Xbox Kinect) on children’s object control skills and found that object control skills improved over time but both studies found no significant differences between the intervention and control groups [27,43]. Notably, the intervention dose of these two studies ranged from 50 to 60 min per week for 6 weeks. In addition, one study examined the effects of an 8-week AVG-based intervention on preschool children’s gross motor skills and competence using the Nintendo Wii and found that there was no significant group effect on children’s motor skills between the experimental and control groups [23]. In terms of the positive findings on FMS, one study included two intervention arms with AVG-based and FMS-focused PA to improve children’s object control skills as compared to a control group. Children performed the Xbox Kinect and FMS training program for 8 weeks, 30 min per session, twice per week, and it was found that both the AVG and FMS training groups had significant improvements in object control skills after 8 weeks as compared to the control group, but there was no significant difference between two intervention groups [26]. In addition, one study compared the difference of a commercial AVG (e.g., Xbox Kinect) and a purpose-built AVG on children’s locomotor skills and found that children in the purpose-built AVG intervention group had significant improvements in all locomotor skills (i.e., running, hopping, skipping, jumping, and sliding), whereas the commercial AVG control group children had significant improvements in only one locomotor skill (sliding) [25].

### 3.4. Physical Fitness (Balance, Agility, and Physical Perception)

A total of five studies examined the effects of AVGs on children’s physical fitness [39,40,41,42] (See Table A1). In detail, two studies examined the effects of AVGs on children’s balance, both of which observed a significant improvement in postural stability as compared to a traditional physical education group [40,41]. Notably, both interventions were 6 weeks in duration and around 100 min per week, and one study used iDance whereas the other used Nintendo Wii Fit for their respective AVG interventions. In addition, one study observed significant differences in children’s agility, reaction times, enjoyment, and self-perception of movement competence following a 12-week AVG-based intervention [39]. Another study examined the effects of a child-centered AVG-based intervention on preschool children’s executive functions and perceived physical and social competence and found that the intervention children had significant improvements in executive functions and perceived social competence as compared to a control group [42]. Notably, there was a significant improvement in perceived physical competence between pre- and post-intervention but no significant differences between the two groups was observed. In addition, one study examined the effects of an AVG-based intervention on physical fitness among children with overweight and obesity. The results indicated that the intervention children had significant improvements in their physical fitness, such as reaction time, speed, and agility, compared to the control group [31].

## 4. Discussion

Given the innovative and interactive experience of AVGs, an increasing number of studies have attempted to adopt such technology as an intervention approach to promote FMS among children. As more studies are available, it is important to systematically synthesize these study findings and provide practical implications and direction for future studies in this emerging field of inquiry. Therefore, the aim of this systematic review was to synthesize and review the latest literature examining the effects of AVGs on healthy children’s FMS development and physical fitness. Overall, it appears that AVGs have the potential to improve healthy children’s physical fitness, such as balance, postural stability, and agility, while findings regarding the effects of AVGs on healthy children’s motor skills development are inconsistent and thus warrant further investigation.

A total of 10 eligible studies were located that evaluated healthy children’s FMS via locomotor, object control skills, and physical fitness. Most of them were implemented in the school setting, indicating schools to be the most popular venue for implementing AVG-based interventions for improving healthy children’ FMS and physical fitness. Among the reviewed studies, five reported positive findings on one or more motor skills and physical fitness (e.g., reaction time, agility, and balance) [25,26,39,40,41] and four studies reported non-significant group differences in children’s FMS [23,27,42,43]. Notably, no study observed negative effects.

Overall, the effectiveness of AVGs on children’s FMS and physical fitness remains inconclusive, with mixed findings. However, preliminary evidence suggests that AVGs consistently have positive impacts on children’s postural stability and balance skills. Based on the reviewed studies, findings regarding object control skills are mixed. In detail, one study implemented an 8-week AVG-based and traditional PA training program (30 min per session, 3 days per week) as compared to a non-treatment control group which examined object controls skills over time and between groups [26]. Their findings indicated that there were significant improvements in object control skills in both intervention groups. Moreover, children in the AVG group reported higher enjoyment compared to the traditional PA training group. Notably, there was no significant difference between the two intervention groups regarding object control skills. However, two studies examining the effects of AVG-based interventions on children’s object control skills reported non-significant differences between intervention and control groups [27,43]. Notably, both studies indicated that intervention duration (both studies were 6 weeks) and dose (50–60 min per session, once per week) may not have been sufficient enough to observe significant changes. Notably, both studies reported that children’s previous AVG experience (or having an AVG system at home) was a confounding factor for object control skill competence at baseline. For example, one study observed a positive association between AVG ownership and FMS, such that children who had AVG systems at home had greater FMS proficiency, which may mask the motor skills improvements following AVG-based interventions because they are able to practice at home and achieve relatively higher FMS than children who do not own AVGs. Indeed, previous findings have indicated a positive association between time spent playing AVGs and better object control skills [44]. Therefore, it is important to address baseline AVG experience when conducting an AVG-based intervention among children.

In regard to the locomotor skills, there was one study that strictly targeted this outcome [25]. In detail, the study examined five locomotor skills in children (e.g., running, hopping, skipping, jumping, and sliding) between a commercial AVG (e.g., Xbox 360 and Wii) and a custom, purpose-built AVG. The results indicated that children in the purpose-built AVG group had significant improvements in all five locomotor skills while children in the commercial AVG group had significant improvement in only one locomotor skill (sliding). The findings revealed a potential issue regarding the implementation of commercial AVGs. Specifically, because the commercial AVGs (e.g., Xbox Kinect, Wii, PlayStation) are not designed specifically for the purpose of improving FMS, some players may have poor-quality outputs during game play which prevent improvements in players’ FMS outcomes. Indeed, while the commercial AVG deployed in this study observed post-intervention improvements in overall locomotor skills, there were only minimal improvements across the individual locomotor skills of running, hopping, skipping and jumping. This is consistent with previous findings that commercial AVGs may allow users to perform poor-quality, or cheated, movements and still achieve success in the game [45]. For example, one Xbox Kinect game named “400m hurdles” requires players to take a proficient jump for passing the hurdles as soon as possible. In order to finish faster, some children may perform small, poor-quality jumps with poor biomechanics (e.g., no use of the arms) instead of a full, maximal-effort jump. In contrast, the purpose-built AVG was intentionally developed with adaptable features allowing a teacher to personalize the players’ playing experiences by manipulating in-game features, such as scoring and timing systems and targets. The study implemented the “human-in-the-loop” approach to manipulate the AVG context to be more appropriate for and specific to children’s FMS development. In other words, a teacher became a part of the AVG intervention to supervise and manipulate the gaming environment to meet the standards for motor skills development. This study emphasized “quality of play” during AVG-based interventions such that a high-quality and consistent AVG playing experience may be a key factor for effectively improving children’s overall FMS. This study further supports the use of AVGs as an effective platform for improving FMS among children and offers new insights into this research line in that AVGs should aim to incorporate a spectrum of FMS components to allow children to practice a variety of movement patterns/skills encompassed in FMS during game play. Moreover, this study included a teacher as an intervention component who manipulated the environments and supervised the intervention process to ensure quality intervention implementation. Compared to the other studies that did not involve a teacher or other research personnel to provide instruction or feedback during the AVG playing, the quality of game play of these interventions may have been compromised and thus may have contributed to the non-significant results.

In addition, Gao et al. in an 8-week AVG-based intervention examined gross motor skills (i.e., locomotor and object control skills) among preschool children and reported non-significant group differences in motor skills competence [23]. Notably, motor skills competence significantly improved from baseline to post-intervention while intervention children demonstrated greater, yet non-significant improvements in FMS. As previously mentioned, intervention duration is a key factor for observing changes in FMS which was also reported in this study to be a limitation in that the relatively short intervention duration (8 weeks) may have hindered improvements in FMS outcomes. Echoing previously reviewed studies that targeted FMS, two studies reported significant improvements [25,26] and three studies reported greater, yet non-significant improvements [23,27,43]. It appears that AVGs have the potential to positively impact children’s overall FMS to some extent; however, the quality of employed AVGs, intervention duration, intervention dose, and participants’ characteristics, such as previous playing experience, need to be carefully considered in the conception of future studies in order to maximize FMS improvements. Based on the current review, the effects of AVG-based interventions on healthy children’s FMS are promising yet remain inconclusive.

Overall, based on the existing evidence, the findings are in favor of AVGs on the positive impacts on children’s physical fitness, such as balance, agility, and physical perception. Four studies examined the effects of AVGs on children’s physical fitness and found significant improvements compared to control groups [39,40,41,42]. Specifically, two studies examined the effects of AVG-based interventions on children’s balance and found significant improvements in postural stability compared to the control groups [40,41]. It appears that the use of AVGs (games which require balancing skills) may effectively improve children’s postural stability. Regarding self-perception, mixed findings were observed between two studies [39,42]. Coknaz et al. examined the effects of AVGs on children’s physical fitness, reaction time, and self-perception and found significant improvements after 12 weeks for all study outcomes [39]. Meanwhile, Xiong et al. compared the effects of a child-centered AVG program and a traditional, teacher-led PA program on preschool children’s executive functions and perceived physical and social competence. The results indicated significant improvements in executive functions and perceived social competence compared to the control group, while children’s perceived physical competence improved over time in both groups, but there were no significant between-group differences. Again, it appears that the intervention duration was an important determining factor. Accordingly, Coknaz et al. implemented a 12-week AVG intervention and observed significant improvements in physical perception, whereas Xiong et al. only adopted an 8-week intervention. Thus, in order to effectively implement AVG-based interventions, an appropriate intervention length is warranted. Overall, the findings on physical fitness, such as agility, balance, and reaction time, consistently showed positive outcomes despite the limited evidence. Findings regarding perceived physical competence and self-perception remain inconclusive.

Although the present study’s major strength lies in the provision of first known synthesis of the most updated empirical evidence regarding the effects of AVGs on healthy children’s FMS and physical fitness, this study is not without limitations. First, the current review only included peer-reviewed full-text and English language publications, despite the fact that other unpublished and non-English studies may be available on this topic. Second, the present study primarily included popular, commercially-available AVGs (e.g., Xbox Kinect, Wii, PlayStation, Dance Dance Revolution) and while there are other types of AVGs, it is possible that the search items used in the current review limited our ability to locate all relevant studies. Third, potential confounding factors, such as gender and age, were not included in the current review. As more high-quality RCTs are available with homogenous FMS assessments, future studies will be able to meta-analyze these outcomes and perform subgroup analyses to provide a more comprehensive synthesis of evidence. Lastly, as a limited number of studies have been included in the current review, our findings should be interpreted with caution.

## 5. Conclusions

In summary, the present study provided an updated review of available high-quality studies examining the effects of AVGs on healthy children’s motor skills development and physical fitness. Based on this evidence, we concluded that AVGs are effective for targeting certain physical fitness components (e.g., balance) in healthy children. Moreover, findings regarding the effects of AVGs on healthy children’s object control and locomotor skills development are inconsistent and thus require further investigation. Regarding the mixed findings on children’s FMS, more studies are needed to draw further conclusions. Given their fun and enjoyable nature, AVGs are promising for the development of FMS in children. However, due to the research design (e.g., lack of studies with follow-up), methodological issues (e.g., lack of AVG session quality control), and intervention dose (e.g., less than 8-week intervention duration), the studies included in the current review demonstrated inconsistent findings of FMS among healthy children. More studies with longer intervention durations are needed to better conclude the effectiveness of AVGs on healthy children’s motor skills development. Based on the limitations of the reviewed studies, the following recommendations are provided for future AVG-based studies:

1. Future studies should select appropriate AVGs that intentionally target children’s locomotor and object control skills.
2. The quality of children’s movement patterns during the intervention period needs to be considered and monitored. Future studies should adopt effective process evaluation measures to ensure the quality of AVG-based interventions.
3. Future studies should adopt appropriate intervention doses, intensity, frequency, and duration. Based on the current review, in order to observe favorable changes, it is recommended to implement an intervention length of at least 8 weeks and at least 30 min per session, 2–3 times per week.
4. In order to generalize the findings, future studies should include a larger and more diverse sample of a variety of schoolchildren.
5. Future studies should consider implementing AVG-based interventions in community- and home-based settings. Based on the current review, none of the studies were conducted in community- or home-based settings, which may have important public implications to assess the effects of AVGs in these settings, especially during the COVID-19 pandemic with the uncertainty of schools reopening.
6. Future studies should include follow-up measures to further examine the long-term effectiveness of AVGs on healthy children’s motor development. Notably, based on the current review, none of studies conducted a follow-up greater than 6 months. It would be informative to investigate the long-term effects of AVG on children’s FMS.
7. Future studies should evaluate participants’ previous AVG experience at baseline as this experience may affect the quality of AVG outputs and movement patterns [46].

## Figures and Tables

**Table 1:** Design quality analysis.

Articles	Randomization	Control	Pre-Post	Retention	Missing Data	Power Analysis	Validity Measure	Follow-Up	Score	Effectiveness
Coknaz et al. [39]	+	+	+	+	−	+	+	−	6	YES
Johnson et al. [30]	+	+	+	+	−	−	+	−	5	NA
Gao et al. [23]	−	+	+	+	+	+	+	−	6	NA
Barnett et al. [27]	+	+	+	+	+	+	+	−	7	NA
Sheehan & Katz [40]	+	+	+	+	+	−	+	−	6	YES
Sheehan & Katz [41]	+	+	+	+	+	−	+	−	6	YES
Vernadakis et al. [26]	+	+	+	+	−	−	+	−	6	YES
McGann et al. [25]	+	+	+	+	+	−	+	−	6	YES
Xiong et al. [42]	+	+	+	+	−	+	+	−	6	NA
Biljon & Longhurst [31]	+	+	+	+	−	−	+	−	5	YES

Note: “+” refers to positive (explicitly described and present in details); “−” refers to negative (inadequately described or absent); “YES” indicates significant positive effect; “NA” indicates no significant effect; median score = 6; retention: retaining more than 70% of the participants; follow-up: following more than 6 months after experiment.

## Appendix A

**Table A1:** Descriptive characteristics of included studies.

Authors	Study Purpose	Sample Size/Settings	Outcomes	Measures	Duration	Intervention	Control	Findings
Coknaz et al. [39]	To examine the effects of AVG on children’s physical fitness, reaction times, self-perception, and enjoyment levels.	*n* = 106; aged 8–12 years school; Turkey	Reaction time; Self-perception	Reaction time was measured by timer as response time to visual and auditory stimuli	12 weeks	Children alternatively played various Wii AVGs that included sports, balancing, aerobic, training, racing games; 50–60 min per session, 3 days per week.	Children in control group maintained their usual school routine.	AVG group showed significant improvement in agility and reaction times and physical perception as compared to control group.
Johnson et al. [40]	To determine whether playing sports AVGs has a positive influence on young children’s actual and perceived object control skills.	*n* = 36; aged 6–10 years school; Australia	Object control skill; Perceived movement skill competence	The Test of Gross Motor Development-3 assessed object control skill. The Pictorial Scale of Perceived Competence for Young Children assessed perceived object control skill.	6 weeks	Children played Xbox Kinect games (e.g., Kinect Sports Season 1, Kinect Sports Season 2, and Sports Rivals); 50 min, once per week.	Children in control group maintained their usual school routine.	No significant differences between the control and intervention groups were observed for both outcomes.
Gao et al. [23]	To examine a school-based exergaming intervention’s effect on preschool children’s perceived competence (PC), motor skill competence (MSC), and physical activity versus usual care (recess).	*n* = 65; aged 3–5 years school; US	Perceived physical competence; Motor skill competence	The Test of Gross Motor Development-2 was used to assess FMS; Pictorial Scale of Perceived Competence was used to assess perceived physical competence.	8 weeks	Wii or Xbox Kinect; 100 min/week AVG session Children had free of choices for single or group playing from Wii, Xbox Kinect, Nickelodeon Fit, and Just Dance for Kids in a large room at school; 20 min per session, 5 days per week.	Children in the control group maintain their weekly 100 min school usual care recess.	There was significant FMS improvement in intervention children but there is no significant difference as compared to control group.
Barnett et al. [27]	To investigate the impact of playing sports Active Video Games on children’s actual and perceived object control skills.	*n* = 95; aged 8–10 years Laboratory; Australia	Object control skill; Object control competence	The Test of Gross Motor Development-2 was used to assess object control skills. The Pictorial Scale of Perceived Movement Skill Competence assessed perceived object control skill.	6 weeks	Children were paired in groups playing selected Wii AVGs; One hour per session, once per week.	Children in control group maintained their usual school routine.	Object control skill improved over time, but there was no significant difference between groups in skill improvement
Sheehan & Katz [41]	To examine the effects of exergaming on children’s balance.	*n* = 64; aged 9–10 years school; Canada	Balance	Postural stability was assessed by the HUR BT 4 Platform, a portable assessment device for testing the postural stability.	6 weeks	Children played in groups on iDance AVG dancing games; 34 min per day; 4–5 days per week	(1) PE with focus on agility, balance, and coordination improvement; (2) Typical PE class	Postural stability significantly improved in exergaming group compared to typical PE class
Sheehan & Katz [42]	To examine the effects of exergaming-based school PE on children’s balance improvement	*n* = 67; aged 8–9 years school; Canada	Balance	Postural stability was assessed by the HUR BT 4 Platform, a portable assessment device for testing the postural stability.	6 weeks	Children group played selected Wii Fit + AVGs that aimed to improve balance (e.g., snowboarding); 34 min per day; 3 days per week	(1) Agility, balance, and coordination PE class; (2) Traditional PE.	Exergaming group children improved postural stability significantly compared to control group (traditional PE); Postural stability was better in girls than boys.
Vernadakis et al. [26]	To compare the difference between exergame-based and traditional object control skills training program among elementary school children.	*n* = 66; aged 6–7 years school Greece	Object control skills	The Test of Gross Motor Development-2 was used to assess object control skills.	8 weeks	(1) Children played Xbox Kinect AVGs (e.g., NBA Baller Beats and Kinect Sports); (2) Children participated in a traditional FMS training program; 30 min per session, Twice per week.	Children in control group maintained their usual school routine.	Significant time effect was found on both exergaming and PA intervention groups; Significant improvement in both exergaming and PA intervention groups, but not in control group; Exergaming group had significantly higher enjoyment than traditional PA group.
McGann et al. [25]	To examine the effects of commercial exergames and purpose-built exergames on children’s locomotor skills.	*n* = 54; aged 5–6 years school; Ireland	Locomotor skills (jump, slide, hop, and skip)	The Test of Gross Motor Development-2 was used to assess FMS.	8 weeks	Children played purpose-built exergame with human-in-the-loop component to manipulate the gaming environment for the development of FMS; 3 min high-intensity session, 5 days per week	Children played on 2 Xbox 360 games and 2 Wii games, such as jump rope, gate keeper; 3 min high-intensity session, 5 days per week	Intervention children had significant improvement in each locomotor skill (run, hop, skip, jump, and slide); Control group had significant improvement in only one locomotor skill (slide)
Xiong et al. [43]	To examine the effects of a child-centered exergaming program and a traditional teacher-led PA program on preschoolers’ executive functions and perceived competence	*n* = 60; aged 4–5 years school; China	Executive functions; Perceived physical competence		8 weeks	4 AVG systems were set up in a room. Children had choices to single or group play during the AVG session (e.g., Nickelodeon Fit, Just Dance for Kids, Wii sports) 20 min per session, 5 days per week	Unstructured recess; 20 min per day, 5 days per week.	Intervention children had significant improvement in executive functions and perceived social competence compared to control group; Significant improvement in perceived physical competence between pre- and post- test, but no significant difference between groups over time.
Biljon & Longhurst [31]	To examine the effects of AVG on overweight and obese children’s physical fitness	*n* = 31; aged 9–12 years; School; South Africa	Motor proficiency	Bruininks-Oseretsky test for motor proficiency was used to test physical fitness	6 weeks	Participants played selected Wii games; 30 min per session, 3 days per week	(1) Participants in this group had access to traditional video games; (2) Participants in this group maintain usual routine.	Intervention children had significant improvements in physical fitness.

Note: AVG, active video game; FMS, fundamental motor skill.
